# Coronary Venous Air Embolism in Placenta Accreta Spectrum Cesarean Hysterectomy

**DOI:** 10.1097/og9.0000000000000067

**Published:** 2025-03-06

**Authors:** AnneMarie Opipari, Jocelyn Spizman, Jourdan Triebwasser, Shitanshu Uppal, Mark Russell, Carlo Pancaro

**Affiliations:** Department of Obstetrics and Gynecology, the Department of Pediatric Cardiology, and the Department of Anesthesiology, Michigan Medicine, and the University of Michigan Medical School, Ann Arbor, Michigan.

## Abstract

**BACKGROUND:**

Placenta accreta spectrum (PAS) is a disorder of placental–uterine attachment often linked to placenta previa and prior cesarean delivery and characterized by hemorrhage with placental separation. Standard treatment for PAS in the United States is cesarean hysterectomy. *Venous air embolism* is a cesarean delivery complication defined as air trapped in the venous system.

**CASE:**

We report a coronary artery venous air embolism during a cesarean hysterectomy managed with a surgical intermission, with mortality risk assessment and temporary conservative management as a bridge to hemodynamic stability.

**CONCLUSION:**

Venous air embolism must be promptly recognized and treated. Acute cardiac events (eg, venous air embolism) in complex obstetric surgery require a methodical approach and interdisciplinary expertise. Given the increasing incidence of PAS and complex obstetric surgery, it is crucial for physicians to be aware of management strategies.

## Abstract 2

We report a coronary venous air embolism during cesarean hysterectomy for placenta accreta spectrum and describe a systematic approach for management.

**Teaching Points**

- Venous air embolism is common in cesarean deliveries but is infrequently clinically significant.
- Early recognition and management of clinically significant venous air embolism is critical.
- If cardiopulmonary complications occur in complex obstetric surgery, surgical timeout with mortality risk assessment with an interdisciplinary team is a strategy to guide intraoperative management.

*Placenta accreta spectrum* (PAS) is a disorder characterized by an abnormal attachment between the placenta and uterus. Pathologically, it is defined as a partial or complete absence of uterine decidua and clinically by the inability to separate the placenta without hemorrhage or the clinical suspicion for hemorrhage based on visual evidence of high-grade pathology. The incidence of PAS is suspected to be rising, estimated as 0.02% of pregnancies in the 1970s to 0.17% in 2019. This rise is attributed largely to increases in cesarean deliveries.^1^

Cesarean hysterectomy is the standard of care for Federation of Obstetrics and Gynecology (FIGO) grade II–III PAS in the United States.^2^ Cesarean hysterectomy for PAS is a complex surgery that is optimized with interdisciplinary teams.^1^ Even with a prepared team, maternal morbidity is 18 times higher in patients with PAS compared with the general pregnant population.^3^

The most significant risk factor for PAS is placenta previa, characterized by partial or complete coverage of the cervix by the placenta, which carries a compounded risk when combined with history of cesarean delivery.^1^ Placenta accreta spectrum is commonly comorbid with placenta previa and managed with central venous access and exteriorization of the uterus. Placenta previa is also the primary obstetric risk factor for venous air embolism, the most common embolic event in cesarean deliveries.^4,5^ Precordial Doppler studies identified venous air embolisms in 50% of cesarean deliveries, with the incidence rising to 97% in cesarean deliveries with general anesthesia and sensitive study techniques.^10^ Despite this high frequency of venous air embolisms during cesarean deliveries, there is a gap in the literature on venous air embolisms during PAS deliveries. We describe a case of suspected venous air embolism during a cesarean hysterectomy for PAS and our management strategy.

## CASE

A 30-year-old woman, G5P1132, with one prior cesarean delivery was diagnosed with complete anterior placenta previa and suspected FIGO grade III PAS on ultrasonographic examination at 19 5/7 weeks of gestation. The patient had no known cardiopulmonary concerns.

The patient was scheduled for cesarean delivery with anticipated hysterectomy at 35 4/7 weeks of gestation, consistent with Society for Maternal-Fetal Medicine guidelines.^6^ On the patient’s arrival to the operating theater, general anesthesia was administered with an endotracheal tube. Central venous access in the Trendelenburg position was obtained through the right internal jugular vein. Fetal monitoring was reactive throughout anesthesia induction.

Routine cystoscopy with stent placement was performed; the left ureteral stent was unable to be passed. The midline vertical laparotomy was uncomplicated. The primary survey noted surgical grade FIGO IIIC PAS. The uterus was exteriorized, the placenta was ultrasonographically mapped, and a hysterotomy was made with No. 10 scalpel and extended with bandage scissors. Delivery of the neonate was uncomplicated. The hysterotomy was closed with the placenta left in situ. Estimated blood loss from the cesarean delivery was 100 mL.

Before the hysterectomy, the patient had a 60-second run of ventricular tachycardia (VT) followed by spontaneous conversion to sinus rhythm and new hypotension of 58/40 mm Hg treated with intravenous norepinephrine and phenylephrine. Oxygen was increased to 100%. The surgical team stopped operating. The patient’s blood pressure temporarily increased to 140/80 mm Hg. Within 5 minutes, she had a second 60-second episode of VT followed by spontaneous conversion to sinus rhythm with hypotension (80/50 mm Hg). Ventilation settings and rotational thromboelastometry were normal and unchanged. A subsequent electrocardiogram (ECG) revealed T-wave inversion and ST-segment depression in leads II and V_5_. There was no evidence of bleeding.

**Fig. 1.**
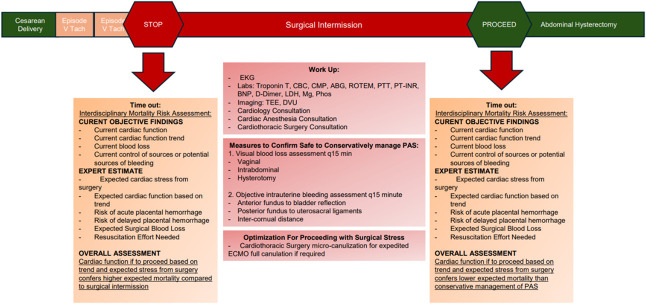
Timeline of surgical events, surgical intermission, and mortality risk assessment. V Tach, ventricular tachycardia; EKG, electrocardiogram; CBC, complete blood count; CMP, comprehensive metabolic panel; ABG, arterial blood gas; ROTEM, rotational thromboelastometry; PTT, partial thromboplastin time; PT-INR, prothrombin time and international normalized ratio test; BNP, B-type natriuretic peptide; LDH, lactic acid dehydrogenase; Mg, magnesium; Phos, phosphate; TEE, transesophageal echocardiogram; DVU, duplex venous ultrasonography; PAS, placenta accreta spectrum; ECMO, extracorporeal membrane oxygenation.

An all-stop timeout was called. Our differential diagnosis, including pulmonary embolism, venous air embolism, amniotic fluid embolism, anaphylaxsis, acute coronary syndrome, and electrolyte abnormality, was reviewed. We completed a primary mortality risk assessment, weighing cardiopulmonary risk with immediate FIGO grade III PAS abdominal hysterectomy against the potential risk of spontaneous placental hemorrhage with a surgical delay (Fig. 1). The consensus was to halt surgical manipulation and complete workup for findings intraoperatively, with the goal of proceeding if an *improvement in cardiopulmonary status*, defined as de-escalation off norepinephrine, was seen. If bleeding presented during the surgical intermission, the team planned to proceed with the hysterectomy with cardiothoracic support on standby for extracorporeal membrane oxygenation (ECMO). A cardiologist was consulted and preformed a transesophageal echocardiogram, revealing an increased right ventricular (RV) systolic pressure of 39 mm Hg, no evidence of septal bowing, and a small patent foramen ovale.

*Opipari. Venous Air Embolism in Cesarean Hysterectomy. O&G Open 2025*.

During the intermission, vaginal bleeding and abdominal bleeding were evaluated every 15 minutes. Serial measurements of defined uterine landmarks assessed for concealed intrauterine bleeding. Urgent laboratory evaluation was completed (Fig. 1). Bilateral lower-extremity duplex venous ultrasonography was negative for deep vein thrombosis. Repeat transesophageal echocardiogram revealed an RV systolic pressure of 30 mm Hg, a hyperdynamic left ventricle, and normal RV function. A repeat ECG revealed sinus rhythm T-wave abnormalities and no residual ST-segment depression. The patient had negative troponins and was weaned off all pressors. A second mortality risk assessment concluded that the risk of spontaneous hemorrhage now carried a higher risk of morbidity than immediate hysterectomy given the interval improvement in cardiac status (Fig. 1).

To optimize potential resuscitation outcomes should cardiac status be compromised with blood loss, the cardiothoracic team recommended microcannulation for expedited ECMO cannulation if needed and placed bilateral femoral catheters. Ultimately, ECMO was not needed.

A total hysterectomy with bilateral salpingectomy was performed. Bilateral parametrial PAS was identified, with left-sided bladder and parametrial involvement causing left urethral tethering and obstruction, requiring reimplantation by urology. Final estimated blood loss was 5,140 mL. Resuscitation included 10 units of packed red blood cells and fresh-frozen plasma, two packs of platelets, 3 g fibrinogen, and 5,000 mL crystalloid.

Immediately after extubation, results of the patient's neurologic examination were normal. The patient recovered in the surgical intensive care unit for 24 hours and then was transferred to labor and delivery. A computed tomography angiogram of the chest was negative for pulmonary embolism. The patient recovered without additional cardiopulmonary concerns.

Our diagnosis is suspected venous air embolism with possible paradoxical coronary air embolism based on clinical presentation, hemodynamic findings, and intraoperative echocardiography, despite the absence of direct air visualization. The sudden intraoperative VT with hypotension, resolving with supportive measures, strongly suggests clinically significant venous air embolism. The patent foramen ovale on transesophageal echocardiogram provided a conduit for paradoxical embolism, allowing venous air to bypass pulmonary filtration. Transiently elevated RV systolic pressure (39 mm Hg, later 30 mm Hg) supports acute right-sided heart strain, consistent with venous air embolism. ST-segment depression (leads II, V_5_) with normal troponins suggests a transient ischemic event rather than infarction, aligning with coronary air embolism. Pulmonary embolism was ruled out by postoperative computed tomography angiography, and electrolyte abnormalities, acute coronary syndrome, and amniotic fluid embolism were excluded. Given the high venous air embolism incidence (50–97%)^7,10^ in cesarean delivery and the patient's risk factors (placenta previa, uterine exteriorization, central venous access, Trendelenburg positioning), we conclude that a transient, hemodynamically significant venous air embolism, with suspected paradoxical coronary embolism attributable to patent foramen ovale, is the most plausible cause of the intraoperative cardiac event.

To manage the suspected venous air embolism intraoperatively, our first step was an all-stop timeout to halt surgical manipulation and to minimize further air entrainment. Because nitrous oxide was not used, we immediately increased oxygen to 100% to enhance nitrogen washout and to reduce the size of any embolized air. The patient was already positioned in left uterine displacement, which we maintained instead of moving to full lateral decubitus both to mitigate further embolization and to allow the possibility of continuing surgery if the patient stabilized. Aspiration through the central venous catheter was attempted but yielded no air. Given the persistence of hemodynamic instability, norepinephrine was initiated to support blood pressure, which improved with intervention. These measures were implemented systematically to stabilize the patient while balancing the need for continued surgical management in a complex obstetric setting.

## DISCUSSION

Venous air embolism is a surgical complication that occurs when air enters the venous system, which can occur during the time of cesarean delivery given exposure of the uterine venous vasculature to outside air. Obstetric risk factors for venous air embolism include placenta previa, uterine exteriorization, central venous catheters, placental abruption, severe preeclampsia, hypovolemia, manual extraction of the placenta, and operation in the Trendelenburg position.^7,8^

Venous air embolism presentation ranges in severity from asymptomatic to clinically significant, with findings of sudden hypotension, tachycardia, hypoxia, hypercarbia, cyanosis, right-sided heart strain on ECG, and dysrhythmias such as VT followed by cardiac arrest.^4,9^ Severity is determined by the rate, volume, duration of air entrapment, and patient's position. Although venous air embolism is common in obstetrics, clinically significant venous air embolism is a rare complication, with a maternal death rate of 1 per 100,000.^7^

Management of intraoperative clinically significant venous air embolism includes flooding the surgical site with saline, ventilating with 100% oxygen, discontinuing nitrous oxide, providing cardiopulmonary support (with volume expansion and inotrope and vasopressor support), aspirating air through central venous catheter, and repositioning (most commonly to left lateral decubitus position).^8^ Prevention of venous air embolism is limited in cesarean deliveries; the focus must be on early recognition and treatment. It is critical that physicians are aware of this vascular complication and respond promptly to prevent further cardiopulmonary destabilization and to direct subsequent intraoperative management.
